# Short-Lasting Unilateral Neuralgiform Headache Attacks with Conjunctival Injection and Tearing in a Patient with Varicella-Zoster Virus Encephalomyelitis

**DOI:** 10.1155/2017/8928017

**Published:** 2017-11-08

**Authors:** Megan E. Gray, Brian Wispelwey, Christopher J. Arnold

**Affiliations:** Division of Infectious Diseases and International Health, University of Virginia, Charlottesville, VA, USA

## Abstract

Short-lasting unilateral neuralgiform headache attacks with conjunctival injection and tearing syndrome (SUNCT) is a type of trigeminal autonomic cephalalgia. Its etiology is generally idiopathic, though rarely it has been associated with viral infections. We describe the fourth case reported in the literature of SUNCT in association with viral meningoencephalitis.

## 1. Introduction

SUNCT is a relatively new disorder, having only first been described in 1978 [1]. There have been few significant epidemiologic studies to date, but one study estimated its incidence rate as low as 1.2/100,000 [1]. It is considered a trigeminal autonomic cephalalgia (TAC) along with trigeminal neuralgia, cluster headache, and paroxysmal hemicrania [2]. The etiology is unknown, and it is generally thought to be idiopathic, although associations with structural intracranial lesions have sometimes been implicated [3]. SUNCT is characterized by unilateral headaches of moderate to severe, stabbing- or sawtooth-patterned pain in the orbital, supraorbital, or trigeminal distribution lasting 1–600 seconds with associated signs of autonomic dysfunction such as rhinorrhea, conjunctival injection, lacrimation, or altered pupillary size of the ipsilateral eye [4].

In a prospective epidemiologic study of patients with SUNCT, only 13.5% of patients had identifiable precipitating events, which included extreme stress; head, facial, or back trauma; viral infection; analgesic withdrawal; and cabin pressure change. In the same study, 6% of patients with SUNCT were also found to have pituitary or posterior fossa lesions, though none were noted to have central nervous system viral infections, such as varicella-zoster virus [3].

Varicella-zoster virus (VZV) is a DNA virus from the alpha-herpes family that is able to stay dormant in dorsal root, cranial, and autonomic ganglia. Reactivation classically leads to the clinical syndrome of herpes zoster, but both primary infection and reactivation can manifest with other neurologic complications including encephalitis, meningitis, myelitis, and stroke, among others [5]. Though there are many known complications of VZV infections in the central nervous system (CNS), the association of SUNCT has only been reported three times in the literature [6–8]. Here, we present a man with SUNCT in association with a confirmed diagnosis of VZV encephalomyelitis.

## 2. Case

The patient is a 74-year-old man with a history of type 2 diabetes mellitus, coronary artery disease, and hypertension who presented with altered mental status. He awoke during the night prior to his presentation with diarrhea and subsequent syncope. Later that morning, the patient was found somnolent in his recliner and a witnessed seizure occurred. Intubation was performed en route to the hospital due to his obtunded state. Basic labs were remarkable for a white blood cell count of 22,000. A computed tomography (CT) scan was significant for air fluid levels in the sphenoid and maxillary sinuses, suggestive of chronic sinusitis. Electrocardiogram and electroencephalogram were unremarkable. Due to his symptoms of headache and altered mental status, a lumbar puncture was performed. Cerebrospinal fluid (CSF) analysis showed a glucose level of 152 mg/dL, protein level of 304 mg/dL, red blood cell count of 9/UL, and white blood cell count of 118/UL (85% lymphocytes, 11% monocytes, and 4% neutrophils). VZV polymerase chain reaction (PCR) was positive in the CSF, and he was diagnosed with VZV meningoencephalitis. Empiric treatment with acyclovir, ceftriaxone, vancomycin, and azithromycin had been started prior to the lumbar puncture. When results returned, the antibiotics were discontinued and acyclovir was continued with recommendations to complete a 21-day course.

On hospital day 2, magnetic resonance imaging (MRI) of the brain and total spine showed an abnormal central cord enhancement from T2 to T5, suggestive of transverse myelitis. Neurologic exam was significant for a sensory deficit across his abdomen (T6 and T7), decreased sensation of the left leg, bilateral lower extremity weakness, diminished reflexes, and bowel and bladder incontinence. On hospital day 5, a repeat MRI of the thoracic spine showed stable findings at T2–T5, but new findings of enhancement of the cord at the T9-T10 level. CSF from a repeat lumbar puncture on hospital day 10 was negative for VZV and herpes simplex virus PCR. His course was also complicated by the development of partial right cranial nerve II and VII palsies.

**Figure 1:**
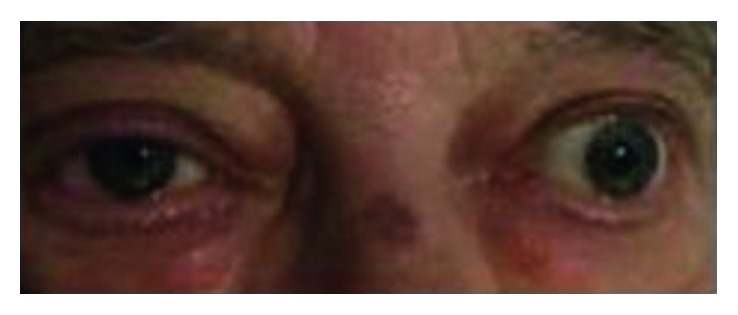
SUNCT-related right eye conjunctival injection and lacrimation are visible, with additional right ptosis related to partial cranial nerve VII palsy.

Additionally, the patient and his family reported a recent history of progressively worsening headaches starting several days before his admission. The headaches were characterized by sharp, stabbing pain around his right eye with radiation to his right ear associated with tearing of his right eye. These headaches were episodic in nature occurring at least 10 times per day, and they continued throughout the initial portion of his admission. Physical exam was also notable for right eye conjunctival injection, tearing, and ptosis (Figure 1). He was seen by neurology, and the overall syndrome was felt to be consistent with SUNCT.

His headaches and dysautonomic symptoms resolved after 4 days of treatment with acyclovir and were controlled with acetaminophen as needed. However, his other neurologic deficits persisted. Treatment with intravenous steroids was subsequently initiated, with isolated, mild improvement in his right leg weakness. He was ultimately discharged to a skilled nursing facility. It is unknown if symptoms of SUNCT have returned as he has not returned to our facility since his discharge.

## 3. Discussion

We report a case of SUNCT associated with VZV encephalomyelitis. There has only been one previous report of SUNCT associated with a confirmed VZV CNS infection in the form of meningoencephalitis. This case occurred in a 72-year-old man who presented with fever and right temporal region headaches. He was originally treated with antibiotics for presumed sepsis, but one week later, he developed right facial nerve palsy in the context of high fever and nausea. A lumbar puncture was then performed, and CSF analysis revealed a positive VZV PCR. He initially improved with ten days of acyclovir treatment but later died after he presented with left-sided hemiplegia and was found to have multiple ischemic lesions on MRI of the brain [6].

Two other cases of SUNCT associated with unconfirmed viral CNS infection are in the literature. The first of these reports described a 46-year-old woman who presented with 1 week of fever and 1 day of altered mental status. She was treated empirically for viral meningoencephalitis with 10 days of acyclovir. VZV IgG and IgM were positive in the serum, but VZV PCR was not performed on the CSF and other studies were negative. An MRI of the brain showed several small hyperintensities on T2-weighted images.

A diagnosis of SUNCT and trigeminal neuralgia was made based on headache symptoms. On day 5 of her admission, she developed partial motor seizures, which were successfully treated with valproic acid. SUNCT headache pain continued for one month after discharge, and the dysautonomic symptoms persisted for six months after discharge [7]. Another case has been reported in a 49-year-old man who presented with fever, typical SUNCT features, and a mild hypoesthesia the distribution of the 1st division of the right trigeminal nerve. His CSF analysis was consistent with viral meningitis, though no PCR testing was documented. This patient was not treated with acyclovir. He was given intramuscular sumatriptan, which was felt to be effective, and his headache symptoms resolved on hospital day 4 [9]. An additional case report detailed a 47-year-old man who developed SUNCT nine days after left ophthalmic distribution VZV, though this patient had no evidence of encephalitis, myelitis, or meningitis [8].

Our patient had multiple complications of a VZV CNS infection including encephalitis, transverse myelitis, and cranial nerve palsies. He also had conjunctival injection and excess tearing that are not commonly seen with the above illnesses. These in conjunction with his headache pattern meet the diagnostic criteria for SUNCT based on the International Headache Society's definition [4]. This would be considered SUNCT secondary to viral encephalomyelitis as any headache in the context of an infection should be attributed to the infection per IHS 3rd edition [4].

SUNCT coinciding with VZV infection has biological plausibility from a pathophysiologic standpoint. VZV can lay dormant in dorsal root, cranial, or autonomic ganglia, and therefore reactivation at any of these ganglia will likely have local effects. VZV is known to reside in the trigeminal ganglia, but should it also reside in the pterygopalatine ganglion which is responsible for innervating the lacrimal gland, tearing and conjunctival injection may ensue with reactivation of the virus [8, 10].

## 4. Conclusion

In summary, we report a case of SUNCT secondary to a confirmed VZV CNS infection in the form of encephalomyelitis. This adds to the small but growing literature on association of SUNCT and VZV infection. VZV infection should be considered in patients presenting with SUNCT.

## Abbreviations

SUNCT: Short-lasting unilateral neuralgiform headache attacks with conjunctival injection and tearing
TAC: Trigeminal autonomic cephalalgia
VZV: Varicella-zoster virus
CNS: Central nervous system
CT: Computed tomography
CSF: Cerebrospinal fluid
PCR: Polymerase chain reaction
MRI: Magnetic resonance imaging.
